# Supplementary material for: Mutations in PCYT2 disrupt etherlipid biosynthesis and cause a complex hereditary spastic paraplegia
Source: Brain. 2019 Oct 22;142(11):3382–97. doi: 10.1093/brain/awz291 (PMC6821184; doi:10.1093/brain/awz291)
Supplement: awz291_Supplementary_Data [file awz291_supplementary_data.zip › awz291-Suppl_data/Supplementary_Data4.zip!/Supplementary information 4 - plasma lipidomics.zip!/Plasma lipidomics/Control vs Patient1/Statistical plots and data/PCA.pdf]

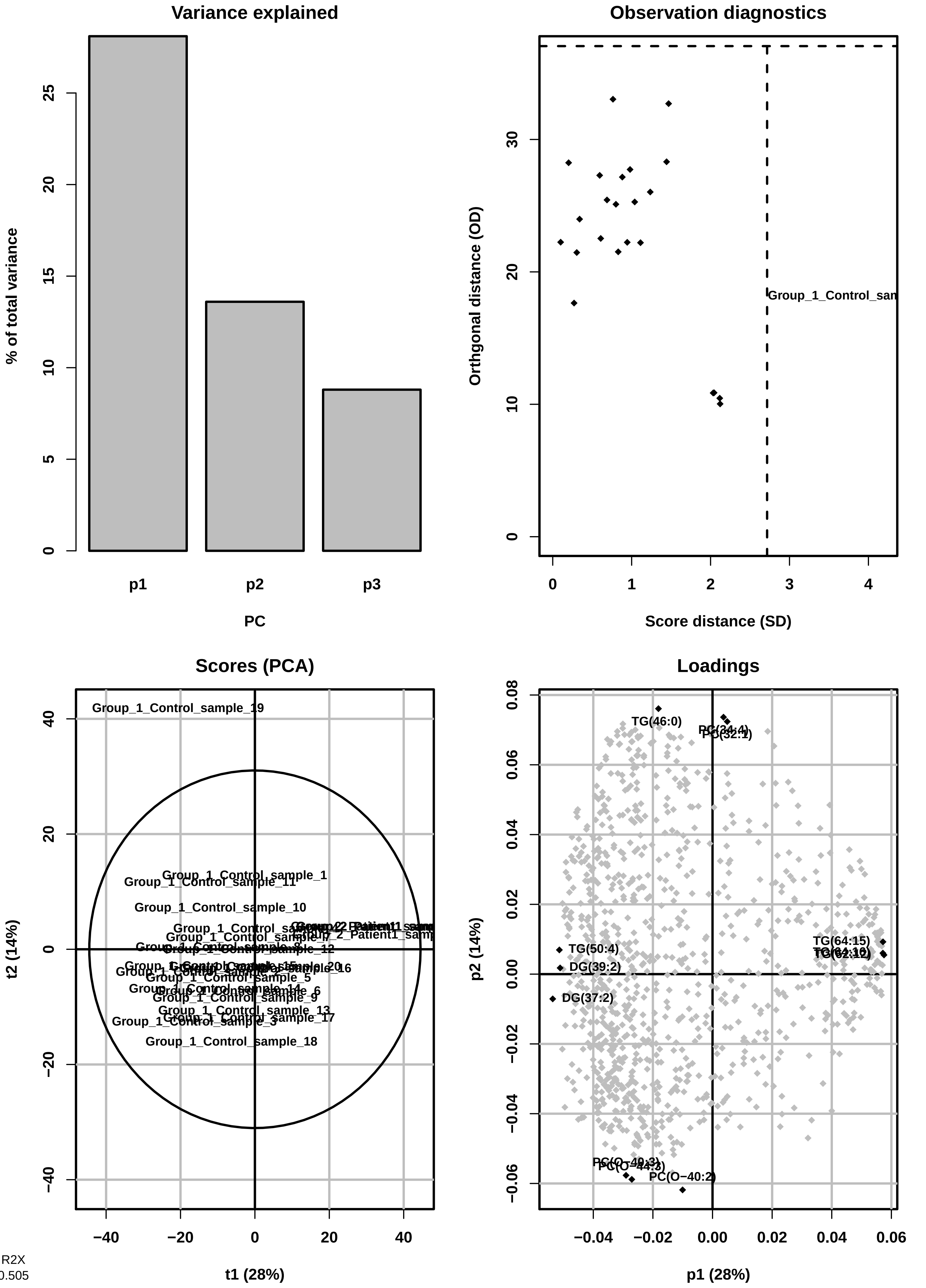
Variance explained
% of total variance
PC
p1
p2
p3
Observation diagnostics
Orthgonal distance (OD)
Score distance (SD)
Group_1_Control_san
Scores (PCA)
t1 (28%)
t2 (14%)
Group_1_Control_sample_19
Group_1_Control_sample_1
Group_1_Control_sample_11
Group_1_Control_sample_10
Group_1_Control_sample_12
Group_1_Control_sample_5
Group_1_Control_sample_9
Group_1_Control_sample_13
Group_1_Control_sample_17
Group_1_Control_sample_3
Group_1_Control_sample_18
Loadings
p1 (28%)
p2 (14%)
TG(46:0)
TG(50:4)
DG(39:2)
DG(37:2)
TG(64:15)
PC(O−40:2)
R2X
0.505
